# Body Mass Index and Its Change from Adolescence to Adulthood Are Closely Related to the Risk of Adult Metabolic Syndrome in China

**DOI:** 10.1155/2021/8888862

**Published:** 2021-02-18

**Authors:** Bingyang Liu, Yue Li, Jiamei Guo, Yuting Fan, Ling Li, Ping Li

**Affiliations:** ^1^Department of Endocrinology, Shengjing Hospital of China Medical University, Shenyang 110022, China; ^2^Department of Endocrinology, Tianjin Third Central Hospital, Tianjin, China

## Abstract

**Aims:**

To investigate the influence of body mass index (BMI) and its change from adolescence to adulthood (ΔBMI) on the risk of metabolic syndrome (MetS) in early adulthood.

**Methods:**

We selected 931 students from 12 to 16 years of age in Liaoyang City, China. Ninety-three participants from 18 to 22 years of age with complete baseline data were available for follow-up after 5 years. Statistical analysis determined the relationship of MetS at follow-up with baseline BMI (BMI_b_), ΔBMI, and follow-up BMI (BMI_f_).

**Results:**

ΔBMI was positively correlated with the change of waist circumference (ΔWC), systolic blood pressure (ΔSBP), triglycerides (ΔTG), uric acid, and glycosylated hemoglobin (ΔHbA1c) in follow-up (*p* < 0.05). For every 1 kg/m^2^ increase in BMI_b_, ΔBMI, and BMI_f,_ the risk of MetS at follow-up increased 1.201-fold, 1.406-fold, and 1.579-fold, respectively. Both BMI_b_ and ΔBMI were predictive of MetS at follow-up, with prediction thresholds of 23.47 kg/m^2^ and 1.95 kg/m^2^. The participants were divided by the predicted BMI_b_ and ΔBMI threshold values into four study groups. Interestingly, the group with lower BMI but a higher increase in BMI presented the same metabolic derangements and Mets% of the group with higher BMI but lower Δ BMI.

**Conclusion:**

Both BMI of adolescence and ΔBMI were predictive of MetS and cardiovascular risk factors in adulthood. Control of both variables in adolescents would be more effective in decreasing the risk of MetS in young adults than control of BMI alone.

## 1. Introduction

Metabolic syndrome (MetS) includes a group of conditions, central obesity, impaired glucose tolerance, hypertension, and abnormal lipid metabolism that are associated with insulin resistance and increases the risk of cardiovascular diseases [1, 2]. The occurrence of MetS and type 2 diabetes in young adults has been increasing along with an increase in obesity among adolescents [3, 4]. To “halt the rise in diabetes and obesity” in adults and children was one of the global health targets set by the World Health Assembly in 2013 [5]. Studies conducted in the west have reported that childhood obesity increased the risk of metabolic diseases in adulthood and that an increase in the body mass index (BMI) from childhood through adolescence was correlated with the development of MetS as an adult [6–8] and was positively associated with early adult ischemic stroke [9]. However, the consequences of adolescent obesity are less well appreciated than those of adult or childhood obesity. That is the case in Asia, and especially in China, where more attention has been paid to childhood academics than physical health. Analysis of the data collected from 1985 to 2010 by the Chinese National Survey on Student Constitution and Health found that 8.2% of adolescents of 7–18 years were obese and that the prevalence of overweight and obesity in China's 31 provinces was 19.2% [10]. Increasing BMI in adulthood has been associated with an increased risk of cardiovascular disease, type 2 diabetes, MetS, and other chronic conditions [11]. However, adult and adolescent metabolism differ [12], and the effects of obesity or being overweight in the growth development period have on the metabolic characteristics in adulthood are not clear.

In this study, adolescents between 12 and 16 years of age living in Liaoyang City in northeastern China were followed for 5 years to determine the impact of BMI in adolescence and its change from adolescent through adult on the development of MetS and other cardiovascular risk factors in early adulthood. The objective was to provide a theoretical basis for adolescents to control weight and prevent MetS.

## 2. Methods

### 2.1. Study Subjects and Design

In December 2010, a group of 931 middle school and high-school students (53.9% male) between 12 and 16 years of age was recruited in Liaoyang, a medium-sized city in northeast China with a moderate degree of economic development. The study population was selected by stratified cluster sampling. The enrolled students had no history of anemia, diabetes, hypertension, or medication history at baseline or at follow-up. In July 2016, 93 participants (53.7% male), now young adults between 18 and 22 years of age, with complete information were available for follow-up. The baseline clinical characteristics of the follow-up population were not significantly different from the 838 study participants who were not available for follow-up (Table 1). The study was approved by the Medical Ethics Committee of Shengjing Hospital of China Medical University and all participants gave informed consent.

### 2.2. Anthropometric and Laboratory Measurements

We followed the methods of Lin et al. [13]. All participants underwent a physical examination and blood samples were collected for measuring biochemical parameters. Height, weight, waist circumference (the smallest diameter between the iliac crest and the lower rib), and hip circumference were measured by a trained physician before blood sample collection. After sitting quietly for more than 10 minutes, blood pressure was measured twice using a desktop Mercury sphygmomanometer and a 2-minute interval between measurements. The average systolic blood pressure (SBP) and diastolic blood pressure (DBP) were recorded. Venous blood samples were collected from participants at 7:00–8:00 in the morning after a ≥10 h fast. Blood samples were transported to a central laboratory at Liaoyang Diabetes Hospital. Plasma was obtained by centrifugation within 1 hour of collection. Fasting plasma glucose (FPG) was assayed by the glucose oxidase method (Olympus 400, Olympus Optical Company, Japan) within 2 hours of centrifugation. Serum low-density lipoprotein cholesterol (LDL-C), high-density lipoprotein cholesterol (HDL-C), triglycerides (TG), aspartate transaminase (AST), alanine transaminase (ALT), and uric acid were determined by standard enzymatic methods. The plasma for hemoglobin Alc (HbAlc) was sent to the central laboratory at Shengjing Hospital of the China Medical University, Shenyang, China, within 4 h and measured using a high-performance liquid chromatography system with a D-10 hemoglobin testing system (BioRad Laboratories, Inc., Hercules, CA, USA). Some plasma was stored at −80°C for assay of fasting insulin (FINS) by radio-immunoassay (China Institute of Atomic Energy, Beijing, China). The methods and investigators at follow-up were the same as at baseline.

### 2.3. Diagnostic Criteria

MetS was diagnosed at baseline in the 12–16-year-old adolescents using the 2007 International Diabetes Federation (IDF) diagnostic criteria [14]. Pediatric obesity was defined as a WC ≥ 90^th^ percentile [15] and MetS was diagnosed when at least two of the additional following criteria were present: (1) an FPG ≥ 5.6 mmol/L (100 mg/dL) or previous diagnosis of type 2 diabetes; (2) an SBP ≥ 130 mmHg or DBP ≥ 85 mmHg; (3) an HDL-C < 1.03 mmol/L (40 mg/dL); (4) TG ≥ 1.7 mmol/L (150 mg/dL). For subjects >18 years of age, IDF adult diagnostic criteria were used, as described below. At follow-up 5 years later, all subjects >18 years of age, and MetS was diagnosed with IDF adult criteria [16]. Central obesity includes a WC ≥ 90 cm in Chinese men and ≥80 cm in Chinese women. Adult MetS also required two of the following in addition to central obesity criteria: (1) TG ≥ 150 mg/dL, or on its relative treatment; (2) HDL-C < 1.03 mmol/L (40 mg/dL) in men and <1.29 mmol/L (50 mg/dL) in women, or on its relative treatment; (3) an SBP ≥ 130 or DBP ≥ 85 mmHg, or treatment of previously diagnosed hypertension; (4) FPG ≥ 5.6 mmol/L (100 mg/dL), or previous diagnosis of type 2 diabetes.

### 2.4. Calculation Formula

BMI = weight/height^2^ (kg/m^2^); delta BMI (ΔBMI) = follow-up BMI (BMI_f_)−baseline BMI (BMI_b_). Similarly, delta WC (ΔWC), delta SBP (ΔSBP), delta DBP (ΔDBP), delta TG (ΔTG), delta HDL-C (ΔHDL-C), delta LDL-C (ΔLDL-C), delta FBG (ΔFBG), delta uric acid (Δuric acid), and delta HbA1c (ΔHbA1c) were calculated as the values at follow-up minus the values at baseline. The homoeostatic model assessment of insulin resistance (HOMA-IR) = fasting blood glucose (mmol/L) × fasting insulin (*μ*U/mL)/22.5.

### 2.5. Statistical Analysis

Statistical analysis was performed using SPSS 22.0 software for Windows. The values of normally distributed variables were compared by the Kolmogorov–Smirnov test. Normally distributed continuous variables were expressed as means ± standard deviation. Variables not normally distributed were reported as medians and four quantiles. Categorical data were expressed as percentages and compared by the chi square test. Analysis of variance was used for multiple comparison of normally distributed data. The Kruskal-Wallis *H* test was used for multiple comparisons of nonnormally distributed data. The receiver operation characteristic (ROC) curve was obtained to evaluate the performance of BMIb and ΔBMI in identifying MetS prevalence in early adulthood. The optimal cutoff point was identified as the coordinate closest to the *y* intercept (0,1) of the ROC curve and, at this point, the sum of the sensitivity and the specificity was maximum. Diagnostic accuracy was assessed by the area under the curve (AUC). Partial correlation was used to analyze the relationships of BMI, MetS, and other cardiovascular risk factors after 5 years. Multiple logistic regression was used to analyze the association of BMIb and ΔBMI with MetS and MetS components at follow-up. Differences with *p* < 0.05 were considered statistically significant.

## 3. Results

### 3.1. Participant Characteristics

The baseline characteristics as adolescents and follow-up characteristics as young adults are shown in Table 1. The mean BMIb and BMIf were not significantly different, either in males or females. The baseline characteristics of the 93 participants evaluated at follow-up were not significantly different from those of the 838 who were not evaluated (Supplemental Table 1) and indicated a representative result for the whole baseline population.

### 3.2. Effects of BMI_b_ and ΔBMI on the Risk of MetS in Early Adulthood

**Figure 1:**
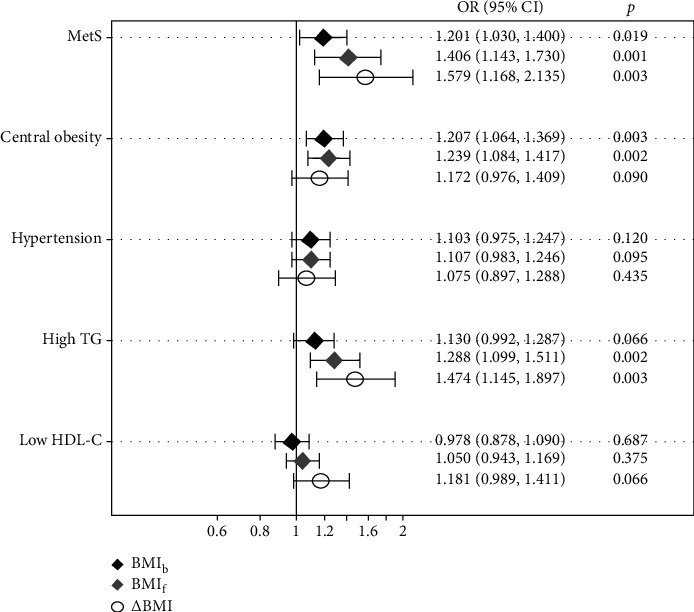
Association of baseline BMI (BMIb), follow-up BMI (BMIf), the change of BMI (δBMI) with the risk of metabolic syndrome (MetS) with central obesity, hypertension, high TG, and low HDL-C at follow up. OR = odds ratio. δBMI = BMIf-BMIb.

After partial correlation analysis to account for the effects of age and gender (Table 2), it was found that ΔBMI was negatively correlated with BMI_b_ and positively correlated with ΔWC, ΔSBP, ΔTG, Δuric acid, and ΔHbA1c (all *p* < 0.05). Therefore, we specifically corrected for the effect of BMIb as a confounding factor in the analysis of the effect of ΔBMI on MetS at follow-up. Logistic regression analysis (Figure 1) showed that for every 1 kg/m^2^ increase in BMIb, the risk of MetS in early adulthood increased by 1.201-fold. The corresponding increases in the risk of MetS were 1.406-fold for BMIf and 1.579-fold for ΔBMI. Analysis of the components of MetS revealed that the risk of central obesity and hypertriglyceridemia at follow-up increased with the increase of BMI_b_ or BMI_f_ (all *p* < 0.05).

### 3.3. ROC Curve Analysis for BMI_b_ and ΔBMI to Predict MetS in Early Adulthood

**Figure 2:**
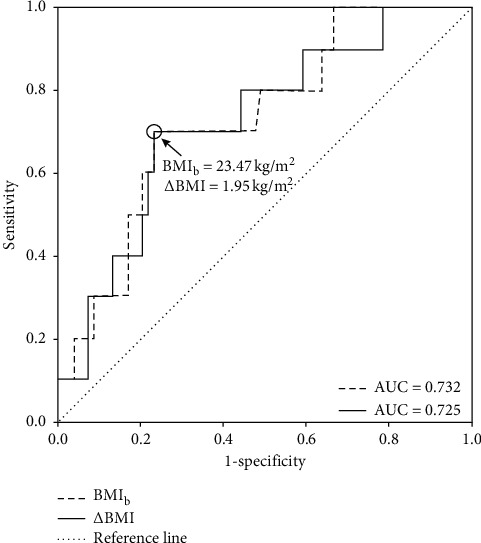
The receiver operation characteristic (ROC) curve for baseline BMI (BMIb) and the change of BMI (ΔBMI) in 5 years to predict metabolic syndrome (MetS) at follow up. AUC, area under the curve; O, the optimal cutoff of BMIb; ΔBMI for identifying MetS.

ROC curve analysis (Figure 2) also found that both BMIb and ΔBMI were predictive of the risk of MetS in adulthood. The AUC values were 0.732 for BMIb and 0.725 for ΔBMI (both *p* < 0.05). The threshold values predictive of MetS were 23.47 kg/m^2^ for BMIb (sensitivity 70%, specificity 77.1%) and 1.95 kg/m^2^ for ΔBMI (sensitivity 70%, specificity 77.1%).

### 3.4. Clinical Characteristics and MetS Risk in Participants Stratified by the Predicted BMI_b_ and ΔBMI Threshold Values

**Figure 3:**
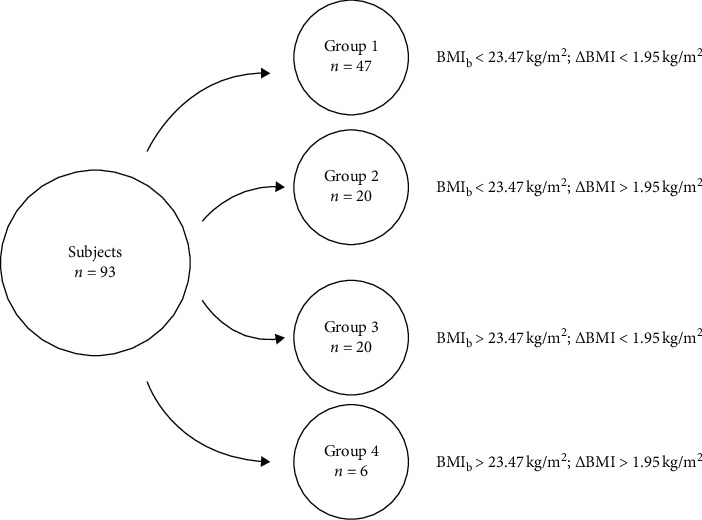
The four groups defined according to baseline BMI (BMIb) and the change of BMI (ΔBMI) in 5 years. BMI, body mass index; BMIb, the baseline BMI; ΔBMI, the change of BMI.

As shown in Figure 3, stratifying the participants by their threshold BMI_b_ and ΔBMI values resulted in four study groups. The groups included those with low BMI_b_ and low ΔBMI (Group 1, *n* = 47), low BMI_b_ and high ΔBMI (Group 2, *n* = 20), high BMI_b_ and low ΔBMI (Group 3, *n* = 20), and high BMI_b_ and high ΔBMI (Group 4, *n* = 6).

The participant characteristics after 5 years were shown in Table 3. Those in Group 1 had the most favorable metabolic characteristics. At follow-up, comparisons between groups with low BMI_b_ found that Group 2 had a higher BMI_f_, WC, TG, HbA1c, and uric acid, but lower HDL-C level than Group 1. Comparisons between groups with high BMI_b_ found that Group 4 had higher BMI_f_, TG, HbA1c, and HOMA-IR than in Group3 (*p* < 0.05). Comparisons between groups with low ΔBMI found that BMI_b_, BMI_f_, WC, SBP, LDL-C, and uric acid were significantly higher in Group 3 than in Group 1. Comparisons between groups with high ΔBMI found that BMI_f_, WC, SBP, TG, and HOMA-IR levels were significantly higher in Group 4 than in Group 2 (*p* < 0.05). Particularly, only WC was different between Group 2 and Group 3, while other metabolic indexes had no statistical difference.

Logistic regression analysis (Table 4) was performed with Group 1 as the reference group with the most favorable metabolic characteristics. The risk of MetS was 5.111-fold higher in Groups 2 and 3 than it was in Group 1. Participants in Group 4 had a 230-fold increased risk of MetS at follow-up than those in Group 1 and a 45-fold increased risk compared with Groups 2 and 3 (all *p* < 0.05).

## 4. Discussion

This study investigated the changes in physical development and metabolic indicators in a group of middle and high school students in northeast China. The participants were 12–16 years of age at enrollment and 18–22 years of age at follow-up 5 years later. We found both BMI level at adolescence and BMI changes from adolescence to early adulthood were predictive of the development of MetS in early adulthood. ΔBMI was positively correlated with the extent of changes in cardiovascular disease risk factors from baseline to follow-up, including the changes of WC, SBP, TG, serum uric acid, and HbA1c. Subjects with higher BMIs in adolescence and larger increases in change of BMI from baseline to follow-up had the highest risk of developing MetS in early adulthood. Compared with participants who had normal BMIs throughout the study, those with either higher baseline BMI or a larger increase in BMI had an increasing trend in the risk of MetS in early adulthood. This result indicated that to reduce the risk of MetS in adulthood, it is equally necessary to avoid a large increase of BMI from adolescence to adult and the control of adolescent overweight, even for adolescents who are not overweight during growth and development.

In contrast to adults, who are concerned with weight control because of the well-documented relationship of BMI and metabolic disease in their age group [17–19], adolescents tend not to pay much attention to the control of their weight. The increase in childhood obesity has prompted studies on the impact of childhood/adolescent weight on the risk of metabolic diseases in adulthood. A cohort study in Spain found that increase in the BMI in a group of boys 5–20 years of age was associated with MetS in adulthood [8]. A subsequent study conducted in Finland found that children who were overweight or obese at 3–18 years of age and remained obese as adults were at increased risk of type 2 diabetes, hypertension, dyslipidemia, and atherosclerosis after 23 years. The risk of those conditions in children who were obese at 3–18 years of age, but were normal-weight adults, was similar to the risk in those who had never been overweight [20]. A recent study that evaluated 62,565 boys who were overweight at 7–13 years found that those who were not overweight as young adults had a significantly lower risk of metabolic disease than those who were still overweight [21]. Another study found that in male adolescents, the increase in BMI was predictive of cardiovascular death in adulthood [22]. The available evidence indicates that both the degree of obesity in adolescence and the amount of change in the BMI from adolescence to adulthood may influence the development of metabolic diseases in adults and warrants further investigation.

In this study, we found that both baseline BMI and ΔBMI in a group of Han Chinese adolescents were associated with MetS in early adulthood. The findings indicate that even in those who are not overweight or obese in adolescence, the increase in BMI during growth and development should be closely monitored. A similar study conducted in adults showed that change in BMI was highly associative with the MetS independently of baseline BMI [23]. The estimated BMI and ΔBMI thresholds may reflect characteristics of adolescents in northeast China that can help guide weight control efforts in that population. The combined use of both threshold values may be more effective than monitoring BMI only. Many previous studies focused on the relationships between overweight and type 2 diabetes, coronary heart disease, or hypertension. MetS comprises several risk factors that are more likely to occur during child and adolescent development than the above single chronic diseases and may more comprehensively reflect the metabolic status of young adults. Stratification of the study population to four groups by the estimated BMI_b_ (23.47 kg/m^2^) and ΔBMI (1.95 kg/m^2^) thresholds was a novel approach that led to the finding that adolescents who were not overweight at baseline but had a ΔBMI above the threshold value could have clinically significant metabolic abnormalities as adults. The results also indicated that control of the increase in BMI could reduce the development of metabolic abnormalities even if in adolescents who had BMI_b_ values above the 23.47 kg/m^2^ threshold. These findings are of great value for the prevention and control of future metabolic diseases in adolescents.

The study limitations include its small sample size, which may have introduced selection bias. Consequently, the applicability of the BMI-related thresholds obtained in this study may be limited. Other limitations include not adjusting the assessment of metabolic characteristics in adolescence by the Tanner scale and a follow-up time of only 5 years, ending in early adulthood. Another limitation could be represented by the difficulty of determining metabolic status in children and adolescents. An increase in the size of the population sample size and extending the follow-up in future studies would help to investigate the relationship of changes in BMI in adolescence and adult diseases.

This is the first study to investigate the impact of BMIb and ΔBMI in adolescents from 12 to 16 years of age in northeastern China during the transition to adulthood on the development of MetS. Both BMI_b_ and ΔBMI were predictive of MetS in early adulthood. Adolescents with a BMI_b_ > 23.47 kg/m^2^ or a ΔBMI > 1.95 kg/m^2^ were at increased risk of MetS in adulthood. Adolescents with a high BMI_b_ and a high ΔBMI had the highest risk of developing MetS. It is recommended that clinicians direct their efforts to control both the BMI and its rate of increase in adolescence with the aim of reducing the risk of MetS in early adulthood.

## Figures and Tables

**Table 1:** Baseline and follow-up clinical characteristics of subjects.

Items	Male (*n* = 50)		Female (*n* = 43)	
	Baseline	Follow-up	Baseline	Follow-up
Age (year)	13.98 ± 1.45	19.98 ± 1.45^*∗*^	14.04 ± 1.31	20.04 ± 1.31^*∗*^
WC (cm)	73.45 ± 9.99	85.78 ± 10.10^*∗*^	70.61 ± 9.47	78 ± 9.27^*∗*^
WHR	0.82 ± 0.06	0.87 ± 0.06^*∗*^	0.78 ± 0.06	0.82 ± 0.14
BMI (kg/m^2^)	22.53 ± 4.08	23.32 ± 3.69	21.6 ± 3.48	22.19 ± 4.09
SBP (mmHg)	122.16 ± 12.60	122.1 ± 10.99	116.91 ± 11.04	112.69 ± 11.04
DBP (mmHg)	71 ± 10.99	80.56 ± 7.72^*∗*^	73.72 ± 10.29	77.20 ± 8.38
TG (mmol/l)	1.03 (0.58, 1.28)	1.29 (0.79, 1.60)^*∗*^	1.01 (0.62, 1.22)	1.03 (0.67, 1.27)
HDL-C (mmol/L)	1.01 (0.84, 1.26)	0.94 (0.85, 1.03)	1.09 (0.94, 1.31)	1.08 (0.94, 1.18)
LDL-C (mmol/L)	3.36 (3.25, 3.46)	2.13 (1.78, 2.55)^*∗*^	3.46 (3.27, 3.54)	2.07 (1.72, 2.36)^*∗*^
FPG (mmol/L)	4.83 ± 0.65	4.14 ± 0.63^*∗*^	4.73 ± 0.51	4.21 ± 0.37^*∗*^
AST (*μ*/l)	19.26 ± 6.57	19.34 ± 12.72	16.60 ± 3.47	17.50 ± 8.25
ALT (*μ*/l)	14.86 ± 10.40	26.85 ± 24.33^*∗*^	11.02 ± 5.61	17.19 ± 14.30^*∗*^
Uric acid (*μ*mol/l)	371.3 ± 85.68	394.25 ± 82.48	277.70 ± 81.70	284.78 ± 87.77
HbA1c (%)	5.43 (5.20, 5.62)	5.2 (5.50, 4.00)^*∗*^	5.44 (5.30, 5.60)	5.37 (5.17, 5.50)
HOMA-IR	4.48 ± 3.39	1.75 ± 0.60^*∗*^	4.28 ± 2.46	1.91 ± 0.78^*∗*^
Obesity (%)	22.00	28.00	23.26	44.17^*∗*^
MetS (%)	14.00	6.00	4.65	16.28^*∗*^

^*∗*^ *p* value <0.05 indicates statistical significance. BMI: body mass index; WC: waist circumference; WHR: waist-to-hip ratio; SBP: systolic blood pressure; DBP: diastolic blood pressure; TG: triglycerides; HDL-C: high-density lipoprotein cholesterol; LDL-C: low-density lipoprotein cholesterol; FPG: fasting plasma glucose; ALT: alanine transaminase; AST: aspartate transaminase; HOMA-IR: homoeostatic model assessment for insulin resistance.

**Table 2:** Partial correlation of the change of BMI and the change of metabolic variables in 5 years.

Characteristic	*r*	*p*
BMI_b_	−0.31	0.01
ΔWC (cm)	0.25	0.04
ΔSBP (mmHg)	0.27	0.03
ΔDBP (mmHg)	0.21	0.08
ΔTG (mmol/l)	0.55	0.00
ΔHDL-C (mmol/l)	−0.17	0.15
ΔLDL-C (mmol/l)	0.23	0.06
ΔFPG (mmol/l)	−0.07	0.57
ΔUric acid (*μ*mol/L)	0.28	0.02
ΔHbA1c (%)	0.30	0.01
HOMA-IR	0.07	0.59

A *p* value <0.05 indicates statistical significance. BMI_b_: the baseline BMI; Δ indicates the change of each variable in 5 years.

**Table 3:** Clinical characteristics of the subjects defined by BMI_b_ and ΔBMI after 5 years.

Characteristic	Group 1 *n* = 47	Group 2 *n* = 20	Group 3 *n* = 20	Group 4 *n* = 6	*p*
MetS (%)	2.12	10.0	10.0	83.3^*∗*^^✚^	<0.001
BMI_b_ (kg/m^2^)	20.2 ± 1.86	20.13 ± 1.86	27.2 ± 3.12^*∗*^^✙^	26.5 ± 3.13^*∗*^^✙^	<0.001
ΔBMI (kg/m^2^)	0.07 ± 1.28	4.12 ± 1.61^*∗*^	−1.75 ± 2.2^*∗*^^✙^	3.48 ± 1.74^*∗*^	<0.001
BMI_f_ (kg/m^2^)	20.13 ± 0.28	24.25 ± 0.56^*∗*^	25.46 ± 0.67^*∗*^	29.99 ± 1.95^*∗*^^✙✚^	<0.001
WC (cm)	77.38 ± 1.23	82.65 ± 1.82^*∗*^	89.95 ± 2.08^*∗*^^✙^	92.33 ± 5.62^*∗*^^✙^	<0.001
SBP (mmHg)	115.04 ± 1.67	115.94 ± 2.76	123.13 ± 2.71^*∗*^	129.8 ± 6.12^*∗*^^✙^	0.009
DBP (mmHg)	78.62 ± 1.22	77.12 ± 2.10	80.13 ± 1.79	85 ± 3.78	0.264
TG (mmol/l)	0.95 (0.66, 1.13)	1.35 (0.09, 1.69)^*∗*^	1.25 (0.74, 1.46)	2.1 (1.69, 2.39)^*∗*^^✙✚^	<0.001
HDL-C (mmol/l)	1.06 (0.94, 1.18)	0.92 (0.82, 0.99)^*∗*^	0.97 (0.83, 1.11)	1.00 (0.91, 1.16)	0.044
LDL-C (mmol/l)	1.97 (1.68, 2.23)	2.19 (1.81, 2.39)	2.26 (1.85, 2.76)^*∗*^	2.45 (2.12, 2.65)^*∗*^	0.025
FPG (mmol/l)	4.20 ± 0.07	4.28 ± 0.14	4.03 ± 0.15	4.12 ± 0.12	0.493
HbA1c (%)	5.23 (5.10, 5.40)	5.44 (5.3, 5.6)^*∗*^	5.17 (5.0, 5.3)	5.5 (5.2, 5.85)^*∗*^^✚^	0.006
Uric acid (mmol/l)	295.3 ± 12.19	383.0 ± 23.39^*∗*^	397.1 ± 19.3^*∗*^	412.5 ± 42.7^*∗*^	<0.001
HOMA-IR	1.63 ± 0.07	1.87 ± 0.16	1.94 ± 0.2	2.76 ± 0.3^*∗*^^✙✚^	0.001

A *p* value <0.05 indicates statistical significance. BMI_f_: the follow-up BMI; △BMI = BMI_f_ − BMI_b_; ^*∗*^*p* < 0.05 compared with group 1; ^✙^*p* < 0.05 compared with group 2; ^✚^*p* < 0.05 compared with group 3. Group 1: BMI_b_ < 23.47 kg/m^2^, ΔBMI < 1.95 kg/m^2^; Group 2: BMI_b_ < 23.47 kg/m^2^, ΔBMI > 1.95 kg/m^2^; Group 3: BMI_b_ > 23.47 kg/m^2^, ΔBMI < 1.95 kg/m^2^; Group 4: BMI_b_ > 23.47 kg/m^2^, ΔBMI > 1.95 kg/m^2^.

**Table 4:** Odds ratios of BMI_b_ and ΔBMI for the follow-up MetS risk after 5 years.

Group 1 (*n* = 47)	Group 2 (*n* = 20)	Group 3 (*n* = 20)	Group 4 (*n* = 6)
1 (reference)	5.11 (0.44, 59.92)	5.11 (0.44, 59.92)	230 (12.38, 4270)^*∗∗*^
—	1 (reference)	—	45 (3.35, 603.99)^*∗∗*^
—	—	1 (reference)	45 (3.35, 603.99)^*∗∗*^

BMI_b_, the baseline BMI; BMI_f_, the follow-up BMI; ΔBMI = BMI_f_-BMI_b_; ^*∗∗*^*p* < 0.01. Group1: BMI_b_ < 23.47 kg/m^2^, ΔBMI < 1.95 kg/m^2^; Group2: BMI_b_ < 23.47 kg/m^2^, ΔBMI > 1.95 kg/m^2^; Group 3: BMI_b_ > 23.47 kg/m^2^, ΔBMI < 1.95 kg/m^2^; Group 4: BMI_b_ > 23.47 kg/m^2^, ΔBMI > 1.95 kg/m^2^.

## Data Availability

The data utilized and analyzed to support the conclusions of our study are included within the article.

## Supplementary Materials

Supplementary Materials Supplemental Table 1: comparison of clinical characteristics between the follow-up subjects and the non-follow-up subjects.
