# Supplementary material for: Body Mass Index and Its Change from Adolescence to Adulthood Are Closely Related to the Risk of Adult Metabolic Syndrome in China
Source: Int J Endocrinol. 2021 Feb 18;2021:8888862. doi: 10.1155/2021/8888862 (PMC7906799; doi:10.1155/2021/8888862)
Supplement: Supplementary Materials — Supplemental Table 1: comparison of clinical characteristics between the follow-up subjects and the non-follow-up subjects. [file 8888862.f1.docx]

Supplemental TABLE 1: Comparison of clinical characteristics between the follow-up subjects and the nonfollow-up subjects.

|  | Follow- up subjects  (n=93) | Nonfollow-upsubjects  (n=838) | *p* |
| --- | --- | --- | --- |
| Age (year) | 14.01±0.143 | 13.71±0.051 | 0.063 |
| WC (cm) | 78.14±1.02 | 76.57±0.37 | 0.172 |
| BMI (kg/m^2^) | 22.10±0.40 | 21.52±0.15 | 0.202 |
| SBP (mmHg) | 119.73±1.26 | 117.36±0.49 | 0.119 |
| DBP (mmHg) | 72.62±1.11 | 73.16±0.38 | 0.653 |
| TG (mmol/l) | 0.94 (0.58, 1.25) | 0.94 (0.68, 1.31) | 0.115 |
| HDL-C (mmol/l) | 1.04 (0.90, 1.26) | 1.07 (0.87, 1.27) | 0.835 |
| LDL-C (mmol/l) | 3.36 (3.27, 3.51) | 3.30 (3.25, 3.48) | 0.113 |
| FPG (mmol/l) | 4.79±0.06 | 4.771±0.02 | 0.836 |
| Uric acid (mmol/l) | 328.02±27.93 | 309.71±3.10 | 0.064 |

A *p* value<0.05 indicates statistical significance.
